# Next‐Generation HER‐2 Tumor‐Targeted Delivery of the STING Agonist Immune‐Stimulating Antibody Conjugate (ISAC) Improves Anticancer Efficacy and Induces Immunological Memory

**DOI:** 10.1002/mco2.70254

**Published:** 2025-07-02

**Authors:** Gang Wu, Chuanfei Yu, Chunyong Ding, Shengtao Yao, Jialiang Du, Zhihao Fu, Yu Liu, Yiming Fan, Guanghao Wu, Ao Zhang, Junzhi Wang

**Affiliations:** ^1^ School of Life Science and Biopharmaceutics Shenyang Pharmaceutical University Shenyang China; ^2^ National Institutes for Food and Drug Control State Key Laboratory of Drug Regulatory Science NHC Key Laboratory of Research on Quality and Standardization of Biotech Products NMPA Key Laboratory for Quality Research and Evaluation of Biological Products Beijing China; ^3^ Shanghai Frontiers Science Center of Drug Target Identification and Delivery National Key Laboratory of Innovative Immunotherapy School of Pharmaceutical Sciences Shanghai Jiao Tong University Shanghai China; ^4^ Shanghai SPH Jiaolian Pharmaceutical Technology Co., Ltd. Shanghai China

**Keywords:** cancer immunotherapy, immune‐stimulating antibody conjugate, immunological memory, STING agonist

## Abstract

Recently, rapidly evolving STING‐based immunotherapies have offered novel therapeutic options for various cancer types. However, systemic administration of STING agonists raises safety concerns, and intratumoral injection is constrained by tumor accessibility. Herein we developed an immune‐stimulating antibody conjugate (ISAC) that links STING agonists to antibodies that target HER2‐positive tumor cells via a cleavable linker. In vivo studies demonstrated that the STING agonist ISAC is well tolerated and exhibits potent antitumor activity in syngeneic mouse tumor models. Investigations in STING‐knockout HER2‐positive tumor cells and STING‐knockout mouse models revealed that the STING pathway primarily mediates antitumor effects upon the activation of immune and tumor cells and that the activation of immune cells plays a stronger role. Additionally, our findings indicate that the STING agonist ISAC enhances both innate and adaptive antitumor immune responses, leading to sustained antitumor activity and the establishment of immune memory. These outcomes support the clinical development of the STING agonist ISACs.

## Graphical abstract

A novel immune‐stimulating antibody‐drug conjugate, which links STING agonists to anti‐HER2 mAb through an optimized noncleavable Linker, remains highly stable at DAR = 5.7 and demonstrates superior efficacy over marketed HER2‐targeted ADCs in multiple tumor models with high, low, and drug‐resistant HER2 expression. And it shows good immune memory against HER2 tumors that have been killed before, thereby achieving rapid re‐killing.

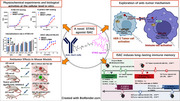

## 1 Introduction

HER2, also known as ERBB2, was identified in 1984 as a member of the EGFR family of transmembrane receptors [1]. Its amplification or overexpression was initially associated with poor survival rates in breast cancer (BC) patients [2], and subsequent research linked HER2 to poor prognoses in other malignancies, including gastric, colorectal, ovarian, and pancreatic cancers [3, 4]. The discovery of HER2 as a sensitive therapeutic target marked a significant breakthrough with the approval of trastuzumab, the first HER2‐targeted monoclonal antibody (mAb), for treating aggressive HER2‐positive BC [5]. Since then, a variety of HER2‐targeted therapies have been developed, including tyrosine kinase inhibitors, single‐epitope mAbs, bispecific antibodies, and recently, antibody‐drug conjugates (ADCs), such as T‐DM1, DS‐8201a, and RC48 [4, 6–9]. Notably, HER2‐targeted ADCs represent a leading‐edge therapy for HER2‐positive tumors, overcoming these limitations by delivering localized antitumor effects through their payloads, thus enhancing therapeutic activity [10, 11, 12, 13, 14]. However, most cytotoxic payloads used in HER‐2 ADC can induce drug resistance and eventually lead to tumor recurrence or metastasis [15]. Therefore, developing novel ADC payload with different antitumor mechanisms and broader therapeutic potential for various solid tumors is critically important.

Modulation of the immune system has emerged as a key strategy in novel cancer therapies, demonstrating potential across various cancer types [16, 17, 18], including the recent exploitation ofTLR‐like receptors (TLR7, TLR8) and STING. TLR7 is mainly expressed in plasmacytoid dendritic cells (pDCs) and B cells, while TLR8 is mainly expressed in monocytes, macrophages and myeloid dendritic cells (mDCs), but not in B cells and pDCs. TLR7/8 activation can induce Th1‐type immune responses and promote the secretion of TNF‐α, IL‐12, and IFN‐α. Studies have shown that TLR7/8 agonists can be used alone or in combination with immune checkpoint inhibitors. They inhibit tumor growth by activating APCs and myeloid cells, promoting T cell infiltration and activation. It is a challenge to ensure that TLR7 and TLR8 agonists can effectively activate the immune response in the tumor microenvironment, while avoiding excessive activation that leads to systemic inflammatory responses and immune‐related adverse reactions. Activation of the innate immune system through signaling pathways such as stimulator of interferon genes (STING) is a promising approach for adjusting the immune response and killing tumor cell [19, 20, 21, 22]. The role of the cGAS‐STING pathway in cancer immunity has been extensively studied in recent years [23, 24, 25, 26, 27, 28, 29, 30, 31, 32, 33, 34, 35, 36]. And now STING has become a hot target for cancer immunotherapy [37]. Preclinically identified STING agonists include cyclic dinucleotide (CDN) derivatives (ADU‐S100, TAK‐676) and non‐CDN molecules such as dimeric amidobenzimidazole (diABZI) [38, 39]. Nevertheless, their clinical value remains uncertain, possibly owing to their restricted administration routes or uncontrollable toxicity related to cytokine release syndrome (CRS). ADCs normally display enhanced pharmacokinetics, reduced systemic toxicity and improved therapeutic indices compared with those of small molecules [40, 41, 42]. Therefore, equipment with a targeted antibody might be an optimal way to achieve tumor selective STING activation [43, 44].

Additionally, Over 50% of HER2‐positive BC patients present with “cold tumors” characterized by deficient tumor‐infiltrating lymphocytes and trastuzumab insensitivity. One of the underlying mechanisms was recently described by Yu and coworkers [45, 46]. Specifically, for the HER2‐positive subtype of BC, trastuzumab resistance and poor prognosis are highly correlated with insufficient STING activation and the expression of the downstream antitumor cytokine CXCL10/11, which are affected by the downregulation of IFI16 (interferon‐gamma inducible protein 16, a cytoplasmic DNA sensor triggering the STING cascade) [45, 47]. While epigenetic modulation of upstream genes such as EZH2/HDACs of IFI16 is one approach, STING agonism for downstream immune activation could be another effective strategy. This phenomenon is unique to HER2‐positive BC, underscoring the rationale for using STING agonism in the treatment of this cancer subtype.

Based on the above analysis, we then constructed a HER2‐targeted STING agonist delivery ISAC with excellent antitumor effects in multiple tumor cell models, and its antitumor effects are superior to those of other marketed HER2‐ADCs (e.g., DS‐8201 and T‐DM1). The antitumor effect of STING agonist ISAC involves the upregulation of type I IFN and other cytokines/chemokines, and we demonstrated that STING plays a synergistic role in tumor cells and immune cells, with immune cells playing a more important role. The results also revealed that the STING agonist ISAC had durable immune memory for the same type of tumor cells with the same type of targeted antigen. STING agonist ISAC could address the limitations of current anti‐HER2 therapies, such as primary insensitivity or secondary resistance. Herein, we present our findings in details.

## 2 Results

### 2.1 Development of a Next‐Generation Anti‐HER2 STING Agonist ISAC

**FIGURE 1:**
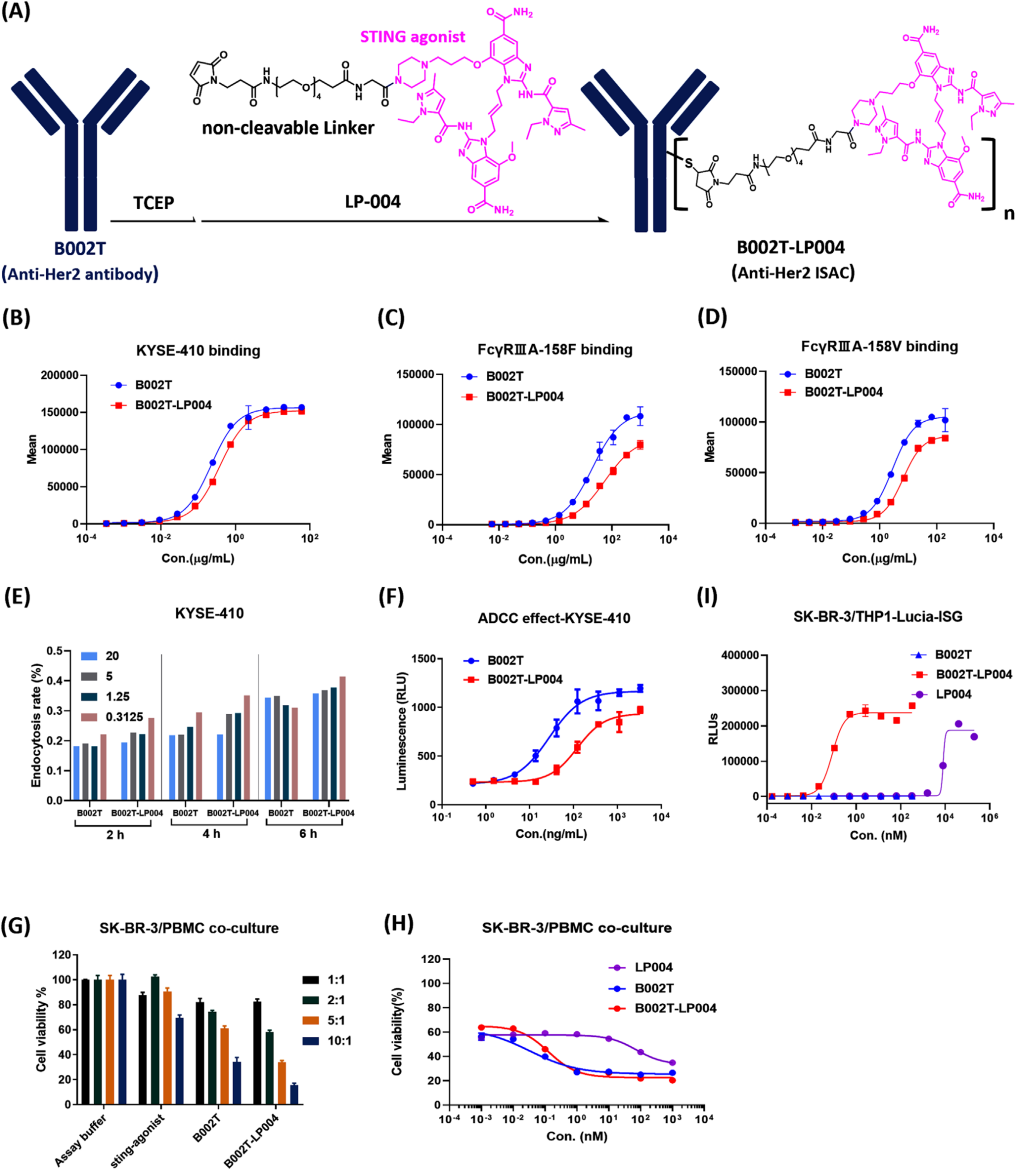
The design of the STING agonist immune‐stimulating antibody conjugate (B002T‐LP004) and its physicochemical experiments and biological activities at the cellular level. (A) Schematic representation of the conjugation of B002T (anti‐HER2 mAb) to LP‐004 (a linker payload containing a STING agonist and a PEG linker). (B) Binding capacity of B002T‐LP004 to the KYSE‐410 cell line, *n* = 3. (C, D) Binding capacity of B002T‐LP004 to FCγRIIIA‐158F and FCγRIIIA‐158 V, *n* = 3. (E) Internalization of B002T‐LP004 in KYSE410 cells. (F) ADCC effect analysis of B002T‐LP004 in KYSE‐410. (G) The activity of B002T‐LP004 at 10 mg/mL in in vitro coculture assays with SK‐BR‐3/PBMC ratios ranging from 10:1 to 1:1. (H) CTG assay for the activity of B002T‐LP004 in the coculture assay at a ratio of SK‐BR‐3 to PBMCs of 5:1. (I) Immunostimulatory activity of B002T‐LP004 in SK/BR‐3/THP‐1‐ISG‐luc cells.

To overcome the adverse effects associated with the use of STING agonists throughout the body and also the drug resistance of anti‐HER2 mAbs, we proposed targeted delivery of the STING agonists to tumor sites by binding to antibodies against HER‐2, which is overexpressed in multiple tumor types. The novel ISAC (named B002T‐LP004) was designed by chemical conjugation of B002T (anti‐HER2 mAb) with LP‐004 (Figure 1A). LP‐004 (consisting of a STING agonist and an optimized noncleavable PEG linker) was developed by our team's previous research work (patent application number of CN 118619928 A). The ISAC`s relevant physicochemical properties have been identified. Figure S1A presents a schematic diagram of all the theoretical conjugation forms of B002T‐LP004 (D0 to D8).

The drug‐to‐antibody ratio (DAR), which represents the average number of drug molecules attached to each antibody, is a crucial parameter for ADCs. First, the DAR value of B002T‐LP004 was determined to be 5.7 via the reduction‐LC/MS method (Figure S1B). Its stability in PBS and plasma after 14 days of incubation was also evaluated, with only 4% and 15% decreases in DAR values, respectively (Figure S1C).

Moreover, both the function of the antibody component and the immunostimulatory activity of the STING component were investigated. The affinity of the Fab region in B002T‐LP004 for the HER2 antigen on the surface of KYSE‐410 cells was slightly weakened compared with that of the naked B002T mAb (Figure 1B), but there was no significant difference between them (Figure S1D, E) when analyzed by an SPR assay using the HER2 antigen fixed in the CM5 chip. In addition, similar to B002T, B002T‐LP004 could also be endocytosed into KYSE‐410 and SK‐BR‐3 cells (Figures 1E and S1F), but the endocytosis efficiency was diminished in the THP endocytosis assay (Figure S1G). Furthermore, the function of the Fc region of B002T‐LP004 was also examined. Compared with that of the naked B002T mAb, the affinity between the Fc regions of B002T‐LP004 and FCγRIIIA‐158F/158 V was lower (Figure 1C, D). The ADCC assay further corroborated the above observation, indicating that the ADCC effect of B002T‐LP004 was less pronounced than that of B002T (Figures 1F and S1H) and that the ADCC function is not the main mechanism of action (MOA) of B002T‐LP004 against cancers.

Next, the antitumor ability of the ADCs was investigated via in vitro coculture experiments. Both B002T‐LP004 and B002T demonstrated clear antitumor effects following huPBMC activation (Figures 1G and S1I). The greater the effect‒target (E/T) ratio is, the more pronounced the drug's effect, with small molecules exhibiting a comparatively weaker impact. The 1:1 and 2:1 E/T ratios resulted in minimal background killing, with the rate of killing being less than 50% after drug action, indicating that the 1:1 and 2:1 E/T ratios were relatively low. A 5:1 E/T ratio was subsequently selected for further investigation of the killing effects of different drug concentrations (Figures 1H and S1J), revealing an intersection point between the two curves of B002T‐LP004 and B002T. At concentrations above the intersection point, the killing effect of B002T‐LP004 was greater than that of B002T, whereas below, the killing effect of B002T‐LP004 was inferior to that of B002T.

The immunostimulatory activity of STING in B002T‐LP004 cells was tested in THP1‐ISG‐luc cells (Invitrogen). B002T‐LP004 induced robust interferon responses with an EC_50_ value of 0.0846 nM, which was more than 1 × 10^−5^‐fold lower than that of the free payload, LP004 (Figure 1I). Unconjugated B002T antibodies did not induce any response, indicating that the stimulation depends on LP004. The immunostimulatory activity of B002T‐LP004 likely cannot be separated from the expression of HER2 on target cells because B002T‐LP004 failed to induce an interferon response in THP1‐ISG‐luc cells lacking HER2 expression (Figure S1K).

Here we constructed an ISAC molecule (B002T‐LP004) and conducted physicochemical and in vitro activity evaluations, including DAR value assay, assessment of the binding ability of the ISAC to HER2, evaluation of its endocytosis efficiency in HER2 target cells, and investigation of its ADCC effect. Compared with similar ISACs, it has a higher DAR value but maintains better stability. In summary, a next‐generation anti‐HER2 STING agonist ISAC was successfully constructed with the predicted properties.

### 2.2 STING Agonist ISAC Exerts Potent Antitumor Effects in Four Cell Line‐Derived Xenograft Mouse Models

**FIGURE 2:**
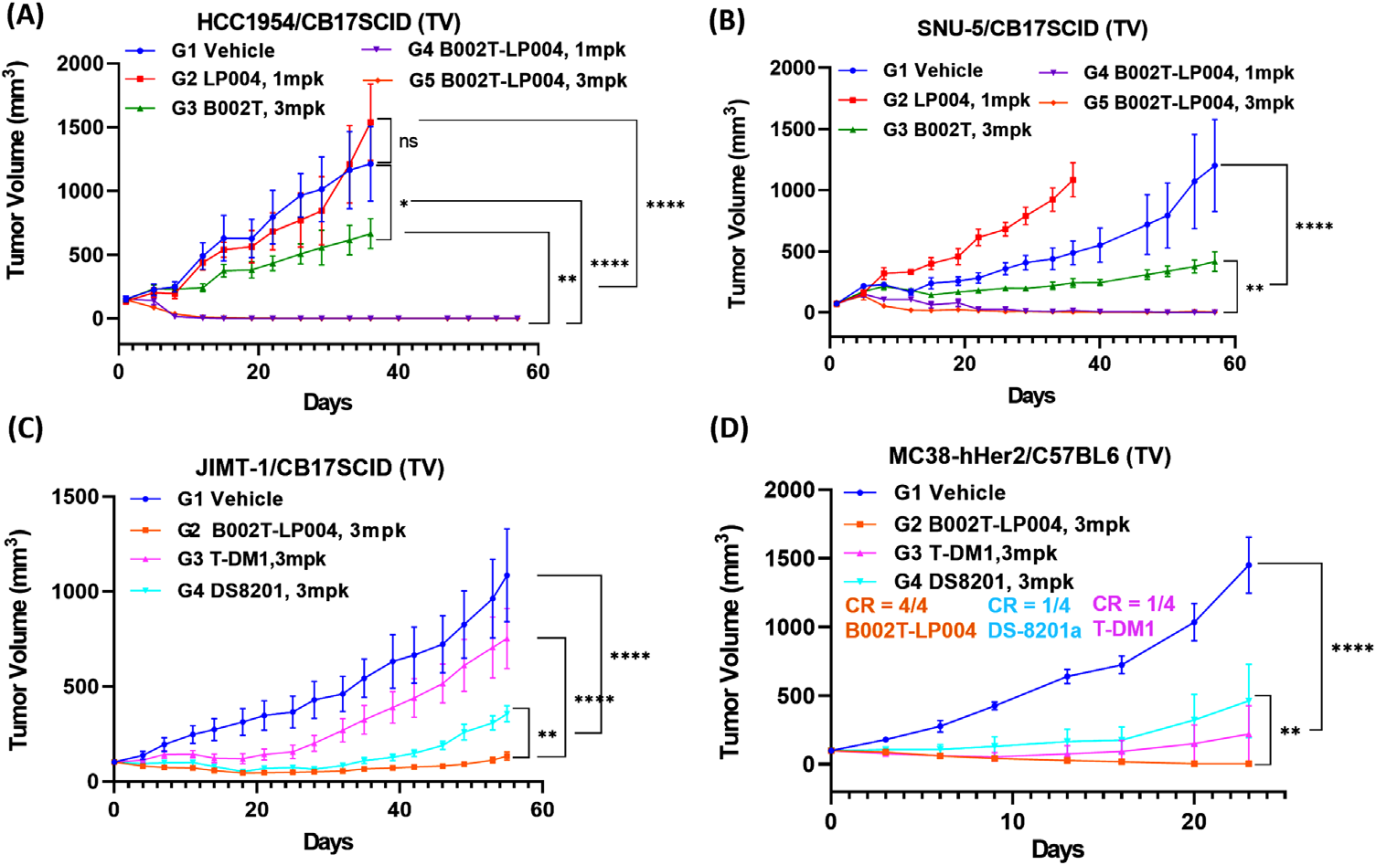
Therapeutic efficacy of B002T‐LP004 in different xenograft mouse models. (A) Mice‐bearing subcutaneous tumors derived from the HCC1954 cell line were treated with storage buffer containing a STING agonist (1 mg/kg), B002T (3 mg/kg) or B002T‐LP004 (1 and 3 mg/kg). (B) Mice‐bearing subcutaneous tumors derived from the SNU‐5 cell line were treated with storage buffer, a STING agonist (1 mg/kg), B002T (3 mg/kg) or B002T‐LP004 (1 and 3 mg/kg). (C) Mice‐bearing subcutaneous tumors derived from the JIMT cell line were treated with storage buffer, B002T‐LP004 (3 mg/kg), T‐DM1 (3 mg/kg), or DS8201 (3 mg/kg). (D) Mice bearing subcutaneous tumors derived from MC38‐hHER‐2 cells were treated with storage buffer, B002T‐LP004 (3 mg/kg), T‐DM1 (3 mg/kg), or DS8201 (3 mg/kg). The average tumor volumes were calculated and presented as growth curves. The data are presented as the means ± SEMs, *n* ≥ 6. Statistical significance was calculated by two‐way ANOVA; ns: no significant difference, *p < 0.05, **p < 0.01, ****p < 0.0001.

For testing the in vivo efficacy of B002T‐LP004, four cell line‐derived xenograft (CDX) mouse models were selected for testing the in vivo efficacy of B002T‐LP004, including the HCC1954/CB17SCID immunodeficient murine HER2 high‐expression human breast cancer model, the SNU‐5/CB17SCID immunodeficient murine HER2 low‐expression human gastric cancer model, the JIMT/CB17SCID immunodeficient murine human breast cancer model, and the MC38‐hHER2/C57 immunocompetent murine colorectal cancer model. B002T‐LP004 demonstrated notable tumor inhibitory efficacy in each mouse model. In both the HCC1954 and SNU‐5 mouse models (Figure 2A, B), B002T‐LP004 demonstrated superior efficacy to the same dose of the B002T mAb. Furthermore, it exhibited superior efficacy to a higher equivalent dose of the small molecule LP004. Notably, B002T‐LP004 exhibited complete tumor suppression at the lower dose (1 mg/kg (mpk)). Interestingly, B002T‐LP004 demonstrated significantly greater potency than equal doses of DS8201 and T‐DM1 in both the HER‐2‐resistant JIMT/CB17SCID immunodeficient murine breast cancer model and the MC38‐hHER2/C57 immunocompetent murine colorectal cancer model (Figure 2C, D). The B002T‐LP004 group of mice exhibited complete tumor inhibition. The above results demonstrated that B002T‐LP004 displayed notable tumor inhibitory efficacy in each mouse model.

### 2.3 STING Agonist ISAC Induces Long‐Lasting Immune Memory

**FIGURE 3:**
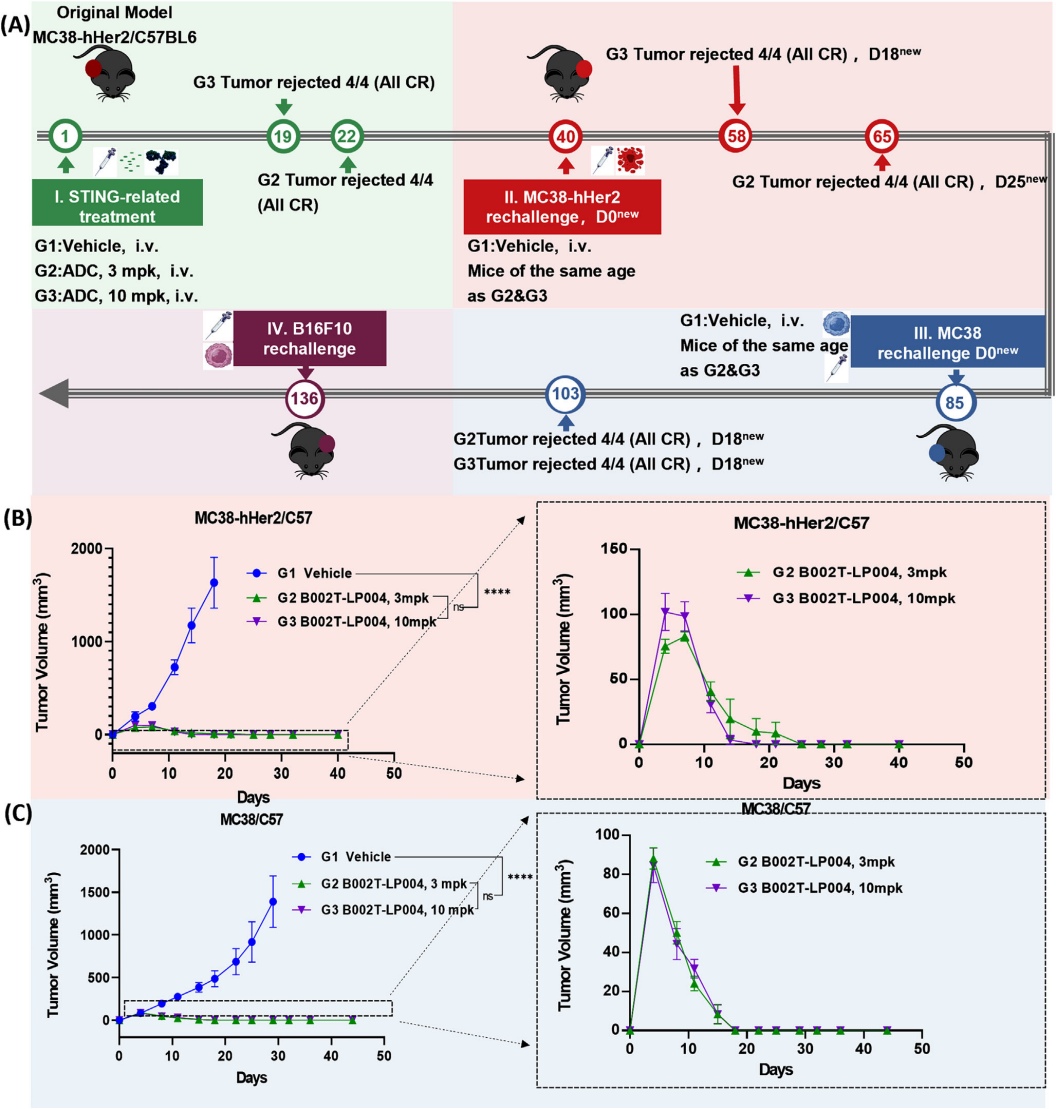
Long‐lasting immune memory function of B002T‐LP004 in mice model. (A) Schematic representation of the immune memory function experiments. (B) wt BALB/c mice or cured B002T‐LP004‐treated mice (*n* = 5/group) were injected subcutaneously with 2 × 10^6^ MC38‐hHER2 strains, and tumor growth was measured and compared twice a week. *p < 0.05, compared with naive wt mice of the same age. (C) wt BALB/c mice or cured B002T‐LP004‐treated mice from (B) (*n* = 5/group) were injected subcutaneously with 2 × 10^6^ MC38 cells, and tumor growth was measured and compared twice a week. The data are presented as the means ± SEMs, *n* ≥ 6. Statistical significance was calculated by two‐way ANOVA; ns: no significant difference, ****p < 0.0001.

Furthermore, we investigated whether B002T‐LP004 can induce immune memory and whether immune memory is B002T‐LP004 specific (Figure 3A). A transplanted tumor model was first constructed via MC38‐hHER2 C57 mice (the tumor cell injection site is marked in red on the left). The mice were subsequently administered 3 mpk and 10 mpk doses of B002T‐LP004, as shown in Figure S2A. Administration of B002T‐LP004 at doses of 3 mpk and 10 mpk resulted in both suppression and regression of tumors. The inoculation of MC38‐hHER2 cells on the opposite side of the cured mice (the tumor cell injection site is marked in red on the right side of the mice) subsequently led to the suppression and regression of tumors in the B002T‐LP004 group, which occurred rapidly after a brief period of growth, as shown in Figure 3B. The growth of the tumor cells rapidly decreased, and B002T‐LP004 inhibited the growth of the same type of tumor cells at the same target site. Following the regression of the MC38‐hHER2 tumor for a second time, the MC38 cells were reinjected into the cured mice (the site of the tumor cell injection is marked in blue on the left side of the mice). As shown in Figure 3C, the MC38 cells of the mice in the B002T‐LP004 group also rapidly regressed following a brief period of growth, suggesting that B002T‐LP004 inhibited the growth of the same type of tumor cells with distinct target sites but not the same type of tumor cells. However, it appears to affect tumor cell growth differently. Finally, the mice that had been cured were inoculated with B16F10 cells once more (the tumor cell injection site is marked in purple on the right side of the mice). Unfortunately, immune cells do not appear to recognize the growth of different types of tumor cells with different targets (Figure S2B).

### 2.4 Exploring the Mechanism of Action of STING Agonist ISAC In Vivo

**FIGURE 4:**
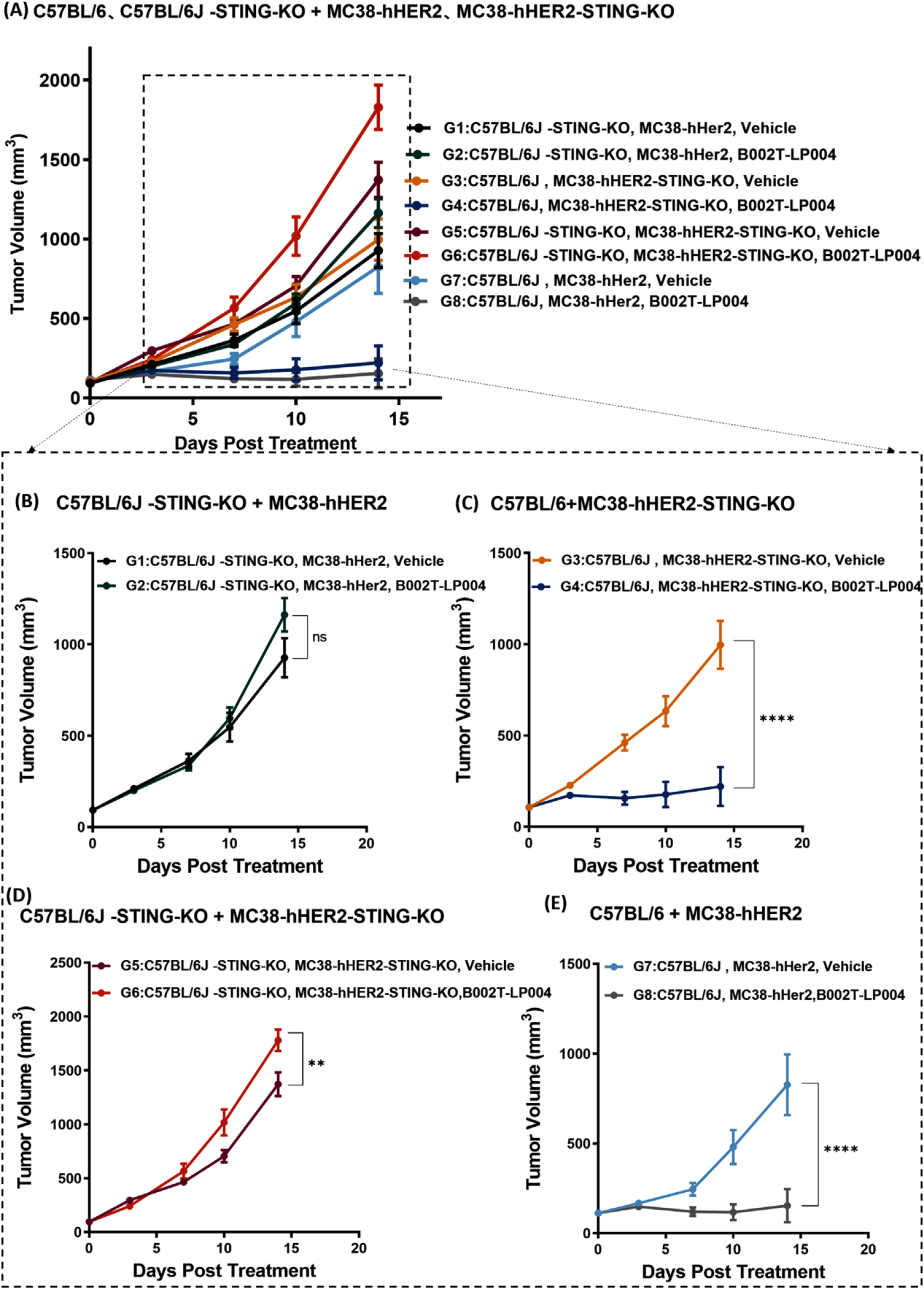
Identification of STING signaling in host and tumor tissues for tumor rejection. (A) Results of tumor growth in four different models. (B) Tumor growth in G1 and G2: C57BL/6J mice with STING knockout bearing MC38‐hHER2. (C) Tumor growth in G3 and G4: wild‐type C57BL/6J mice bearing MC38‐hHER2 STING knockout. (D) Tumor growth in G3 and G4: C57BL/6J mice with STING knockout bearing MC38‐hHER2 STING knockout. (E) Tumor growth in G5 and G6: wild‐type C57BL/6J mice bearing MC38‐hHER2. All the mice were intratumorally treated with vehicle or ISAC when the tumor volume reached approximately 90 mm^3^. The data are presented as the means ± SEMs, *n* ≥ 6. Statistical significance was calculated by two‐way ANOVA; ns: no significant difference, **p < 0.01, ****p < 0.0001.

To explore the in vivo MOA of B002T‐LP004, different transplantation tumor models were established to investigate the potential involvement of both the host and tumor tissue STING pathway in tumor suppression following B002T‐LP004 administration. (The details of the experimental design are delineated in Table S1.) The wild‐type (wt) C57BL/6 mice, C57BL/6J STING knockout (C57BL/6J‐STING‐KO, purchased from GemPharmatech Co., Ltd.), Mc38‐hHER2 cells and MC38‐hHER2 STING knockout (MC38‐hHER2‐STING‐KO) cells (Figure S3A, B) were prepared to establish different transplantation tumor models. Figure 4A shows tumor inhibition in all the groups. Specifically, a comparison of G1 (C57BL/6J‐STING‐KO, Mc38‐hHER2, and Mc38‐hHER2, vehicle) and G2 (C57BL/6J‐STING‐KO, Mc38‐hHER2, B002T‐LP004) tumors revealed that the STING of tumor tissues has only a weak tumor‐suppressing effect in the presence of ISAC (Figure 4B). Compared with G3 (wt C57BL/6J, Mc38‐hHER2‐STING‐KO, vehicle) and G4 (wt C57BL/6J, Mc38‐hHER2‐STING‐KO, B002T‐LP004) tumors, when the ISAC drug is administered to mice in the normal state, the number of tumor cells is significantly suppressed (Figure 4C), and when both STING in mice and STING of the tumor cells are removed, the proliferation rate of the tumor cells is significantly increased (Figure 4D). In the cases of G7 (wt C57BL/6J, Mc38‐hHER2, vehicle) and G8 (wt C57BL/6J, Mc38‐hHER2, B002T‐LP004) with wild‐type mice bearing MC38‐hHER2 tumors, G8 with B002T‐LP004 treatment demonstrated the most effective tumor‐suppressive effect. These results suggest that STING signaling in the host is the main driving force underlying tumor killing when B002T‐LP004 is administered, whereas the STING signaling in tumor tissue plays a weaker role.

**FIGURE 5:**
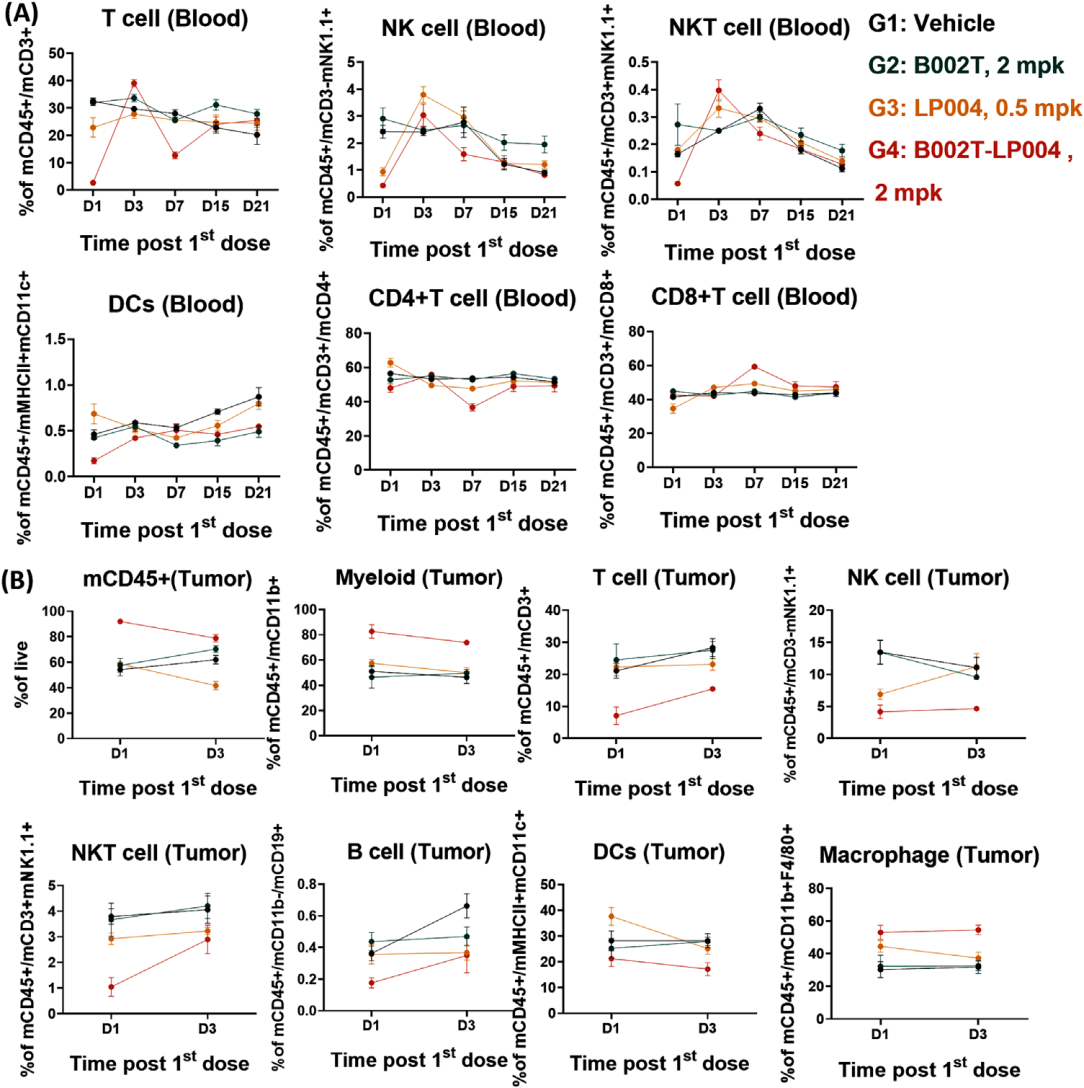
The percentage change in immune cell populations following the administration of various drug agents. (A) Regulation of different types of immune cells in the peripheral blood by the ISAC, as measured via flow cytometry. (B) Regulation of different types of immune cells in tumor tissue by the ISAC, as measured by flow cytometry.

Subsequent to the identification of STING signaling in the host as the principal MOA of tumor rejection, alterations in immune cell populations in the peripheral blood following B002T‐LP004 administration were examined. The specific marker selections for the flow cytometry assay were detailed in Table S2. The results clearly demonstrated that on the first day after B002T‐LP004 administration, the proportion of immune cells in the peripheral blood, including T (CD45^+^ and CD3^+^), NK and NKT (CD45^+^ and NK1.1^+^), DC, Th (CD4^+^), and other cells, rapidly decreased, followed by gradual recovery to a level approaching that of the control group over time. Furthermore, the expression of immune cell activation markers was analyzed, and the results demonstrated that the proportions of activated immune cells, including T, B, NK, NKT, DC, and other cells, were elevated in the peripheral blood (Figure 5A). In light of these findings, we postulate that B002T‐LP004 recruited immune cells from the peripheral blood to the tumor tissue, whereas the remaining T, B, NK, NKT, and other cells in the peripheral blood were still activated by B002T‐LP004 (Figure S4A), IFN‐γ, KC, TNFα, MCP‐1, IL‐12p70RANTES, IL‐1β, IP‐10, GM‐CSF, IFN‐β, and IFN‐α in the peripheral blood of the mice in the ISAC administration group. In addition, the expression of IFN‐γ, KC, TNF‐α, MCP‐1, IL‐12p70, IP‐10, and IFN‐α increased significantly compared with that in the control group, reaching a peak value between 3 and 12 h, followed by a decrease to a level close to that of the control group after 168 h (Figure S4B).

The proportion of CD45^+^ immune cells in tumor tissues was significantly increased following B002T‐LP004 administration compared to the other groups, indicating the upregulation of myeloid cells with inherent natural immunity and increased immune infiltration in tumor tissues (Figure 5B). Conversely, the proportion of lymphocytes, including T, B, NK, and NKT cells, tends to decrease. Additionally, T, B, NK, and NKT cells in the tumor tissue were rapidly activated (Figure S4C), and the trend of cytokine alterations in the tumor tissue was largely consistent with that observed in the peripheral blood (Figure S4D), initially showing an upregulation and subsequently returning to a level comparable to that of the control.

**FIGURE 6:**
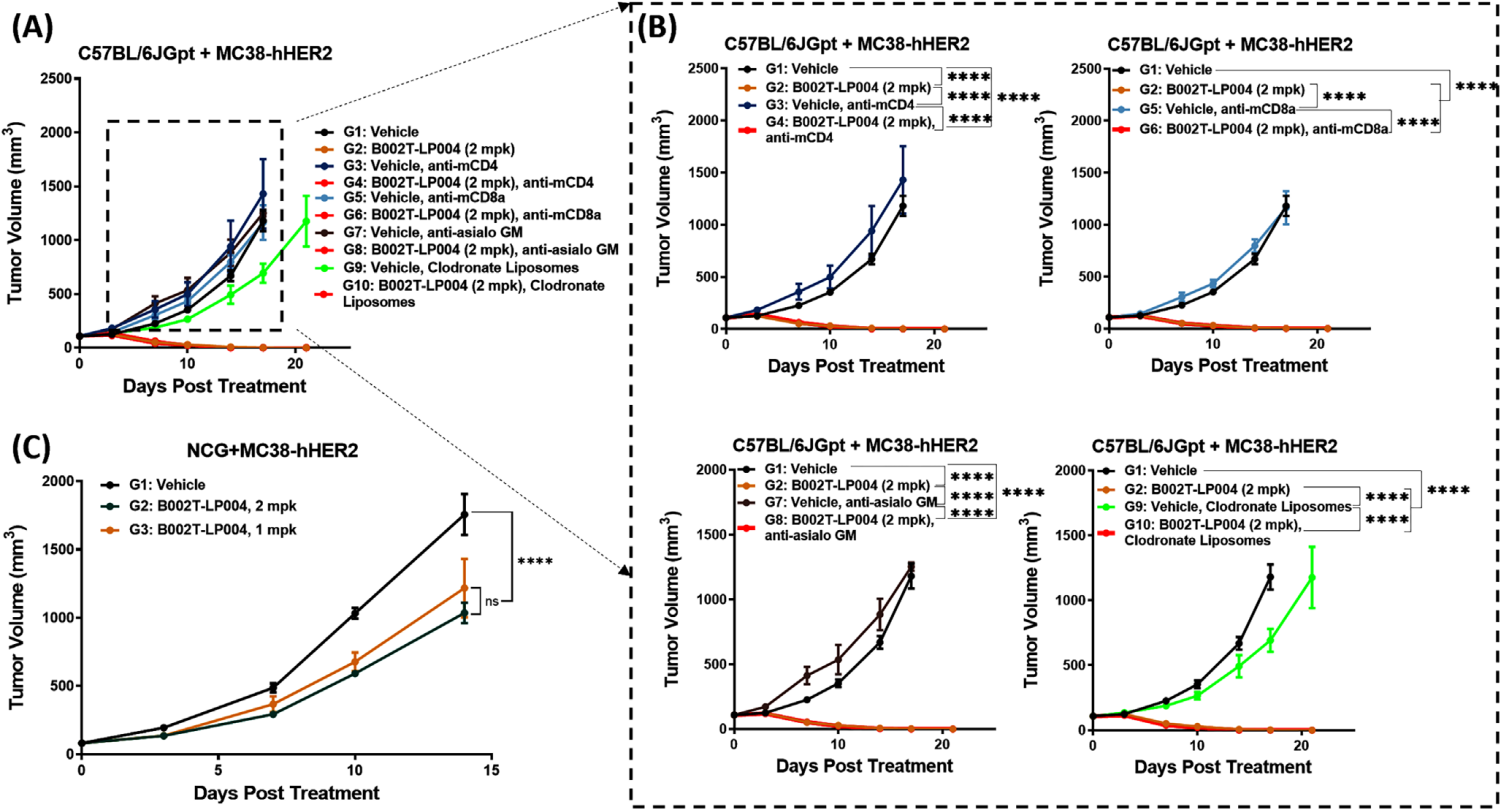
Identification of immune cells involved in STING‐mediated tumor rejection. (A) Tumor growth of MC38‐hHER2 tumor‐bearing C57BL/6J mice treated with ISAC (2 mpk) on day 1; anti‐mCD4, anti‐mCD8 and clodronate liposomes on days 0, 4, 8, 12, 16 and 20; and anti‐asialo GM on days 0, 5, 10, 15 and 20 (*n* = 4). (B) Effect of ISAC drugs on tumor growth after clearance by different immune cells (*n* = 4). (C) Tumor growth in MC38‐hHER2 tumor‐bearing NCG mice treated with ISAC (1 mpk, 2 mpk) on day 0. All the mice were intravenously treated with vehicle or ISAC when the tumor volume reached approximately 80–100 mm^3^. The data are presented as the means ± SEMs, *n* ≥ 6. Statistical significance was calculated by two‐way ANOVA; ns: no significant difference, ****p < 0.0001.

To determine which types of immune cells are activated by B002T‐LP004 during tumor rejection and, thus, which types of immune cells play a more important role in tumor rejection, we first cleared CD4^+^ T, CD8^+^ T, and NK cells and macrophages from C57BL/6jGpt mice bearing Mc38‐hHER2 individually. The mice were subsequently treated with B002T‐LP004 (Table S3), and tumor growth was observed (Figure 6A). The details in Figure 6B demonstrate that the tumor growth rate of the mice that had been cleared of CD4^+^ T, CD8^+^ T, and NK cells was greater than that of the control group initially and that the rate slowed at approximately 15–20 days. In contrast, the tumor growth rate of the mice that had been cleared of macrophages was consistently lower than that of the control group. Unfortunately, it was not possible to draw clear conclusions as to which type of cells played a dominant role in tumor suppression, as in all groups, the tumors regressed rapidly and completely shortly after B002T‐LP004 was given. We subsequently loaded the tumors further and observed the efficacy in NCG (NOD/ShiLtJGpt*‐Prkdc^em26Cd52^Il2rg^em26Cd22^*/Gpt) mice with more severe immunodeficiency. These results indicated that B002T‐LP004 could still exert a tumor inhibitory effect in this model. However, it did not lead to complete regression of the tumor. These findings suggest that B002T‐LP004 may activate macrophages and DCs in NCG mice, thereby inhibiting tumors. In conclusion, it can be hypothesized that B002T‐LP004 likely activates many immune cells when it eradicates tumors. Therefore, the killing activity of B002T‐LP004 can be attributed to the collective action of multiple immune cells.

### 2.5 Safety Profile of B002T‐LP004 ISAC

**FIGURE 7:**
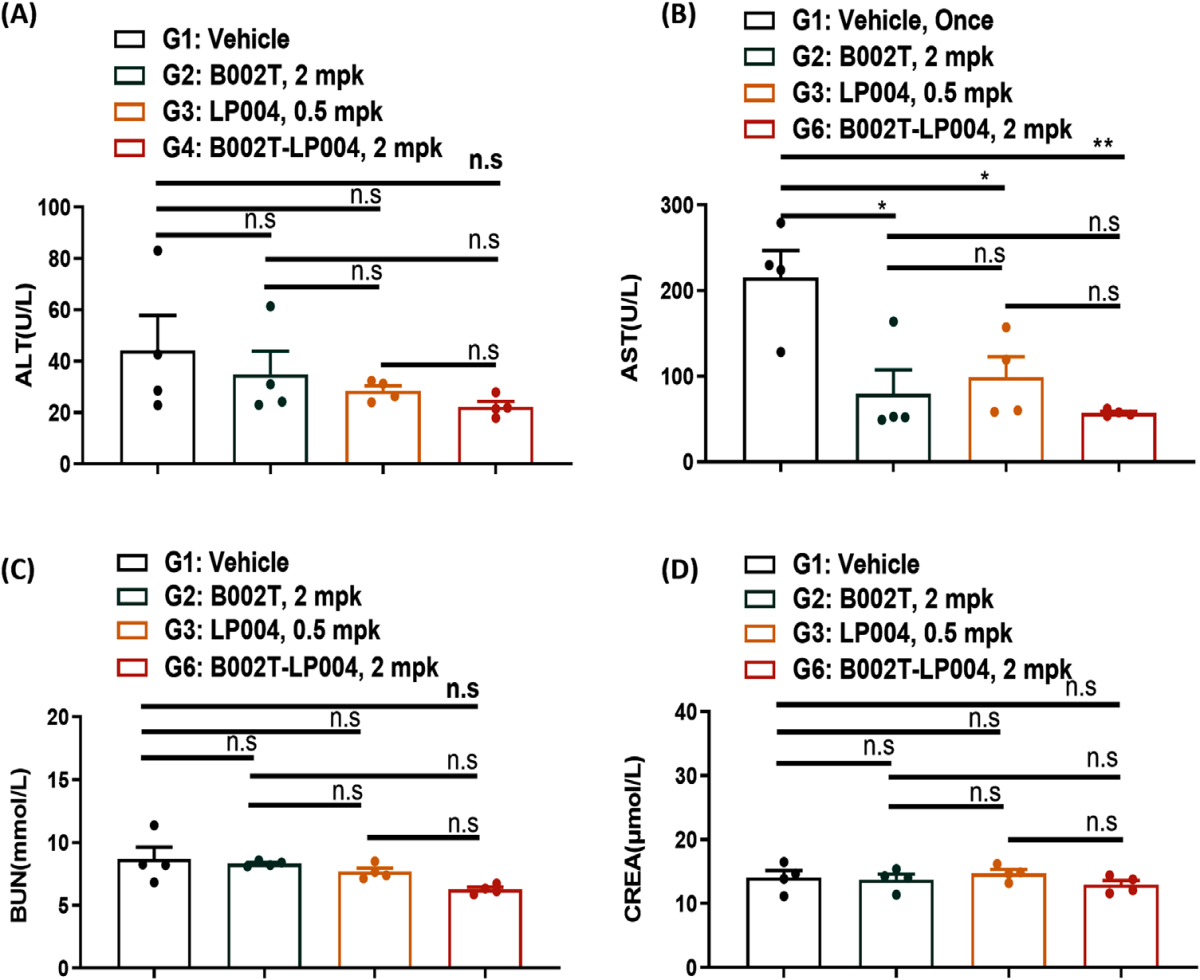
Indicators of hepatic and renal function in mice bearing Mc38 tumors after treatment with ISAC. (A) AST; (B) ALT; (C) BUN; (D) CREA. The data are presented as the means ± SEMs, *n* = 4. Statistical significance was calculated by two‐way ANOVA; ns: no significant difference, *p < 0.05, **p < 0.01.

To evaluate the safety of B002T‐LP004 ISAC, the effects of B002T‐LP004 on the liver and kidney of mice bearing MC38 tumors were examined. First, liver function indices were compared between the B002T‐LP004 and control groups. The B002T‐LP004 group presented a slight decrease in alanine aminotransferase (ALT) and a significant decrease in azelaic aminotransferase (AST) (Figure 7A, B). In this context, the liver indices of healthy mice were also collected (Figure S5A). The ALT and AST indices of healthy control mice were significantly lower than those of the control group because the mice bearing tumor cells had some liver damage. Conversely, B002T‐LP004 administration decreased the ALT and AST indices. With respect to renal function indices, the urea nitrogen (BUN) and creatinine (CREA) levels of the B002T‐LP004‐treated group and the control group did not significantly differ and were close to those of the healthy mice (Figures 7C, D and S5B). Thus, from the results of hepatic and renal indices, our ISAC drug does not appear to exhibit any discernible hepatic or renal toxicity.

## 3 Discussion

The use of HER2‐targeted antibody drugs has resulted in notable advances in the treatment of diverse cancers [48, 49, 50]. Nevertheless, several challenges remain to unsettled with regard to HER2‐targeted drugs, including the development of resistance, tumor recurrence, and metastasis [51, 52]. It is, therefore, highly important to develop new drugs that target HER2 to solve these problems. The development of STING agonists is also being promoted, but progress is not optimistic. Small molecules of STING agonists in the clinical stage are administered by intratumoral injection [53, 54, 55, 56], which greatly limits the scope of their application. Consequently, there is a need to develop STING agonist drugs with a targeting function. The preparation of ISAC drugs by coupling anti‐HER2 antibodies with STING agonists is expected to achieve synergistic enhancement. On the one hand, the anti‐HER2 antibody can still bind to the HER2 antigen, thereby exerting an additional antitumor effect, theoretically preventing HER2 from inhibiting the activation of the STING pathway to a certain extent. On the other hand, the STING agonist introduced into the intracellular space by the antibody can directly activate the STING signaling pathway, thereby killing the HER‐2‐positive tumor cells.

The stability of ADC drugs is an important issue. The shed small molecule drugs are not only toxic but also have a significant impact on pharmacokinetics and efficacy [57, 58]. Similarly, this challenge should also be addressed for ISAC comprised STINGs, as the prematurely detached STINGa can activate STING, triggering adverse reactions in a nontargeted manner. With respect to the in vitro stability of the ISAC, in the preparation solution, the shedding rate of small molecules was only approximately 3% throughout 30 days, mainly due to the design of a hydrophilic‐optimized noncleavable linker of the ISAC. According to the information described in this patent (International Publication Number: WO 2023/109942A1), the shedding rate of the similar product XMT‐2056 in plasma at 48 h nearly 40%, whereas our ISAC was approximately 15%, showing obvious advantages. Meanwhile, the lower shedding rate and the relatively higher drug loading capacity compared to similar drugs lay the foundation for good efficacy.

The expression level of HER2 antigen is crucial for the efficacy of targeted drugs. Natural low HER2 expression or mutations leading to decreased HER2 expression are important reasons for the nonresponse or resistance of targeted drugs [59, 60]. The development of ISAC by us has shown excellent effects not only in the HCC1954 human breast cancer model with high HER2 expression, but also in the SNU‐5 human gastric cancer model with low HER2 expression. Furthermore, B002T‐LP004 exhibited superior efficacy in a trastuzumab‐resistant JIMT human breast cancer model and the murine colorectal cancer model (MC‐38) compared to T‐DM1 and DS‐8201. These results demonstrated that B002T‐LP004 has potent antitumor activity and can address the problem of resistance to anti‐HER2 mAbs.

STING‐ISAC has the ability to activate the immune memory of the body, which has been reported before [43, 44]. This is a characteristic that significantly distinguishes ISAC from other anti‐HER2 targeted drugs (including ADC), and this study on immune memory reached similar conclusions to previous studies. Furthermore, we proved that after the tumor cells were eliminated for the first time by activating STING with the ISAC, the ability to recognize and kill antigen bearing cells was enhanced due to the previous STING activation of the overall passive immune system. Therefore, it has a better re‐killing ability against similar tumors (MC‐38‐hHER2, MC‐38‐HER2), but has no killing power at all against nontarget antigen bearing cells (B16F10).

In vitro SK‐BR‐3/PBMC coculture experiments demonstrated that the efficacy of the treatment is contingent upon the concentration of the administered drug. There exists a threshold concentration above which the efficacy of B002T‐LP004 is superior to that of the naked mAb and below which the efficacy of B002T‐LP004 is inferior to that of the naked mAb. This phenomenon can be attributed to two factors. First, as previously discussed, the ADCC effect of the Fc region of the antibody in B002T‐LP004 is less pronounced than that of a naked mAb. Second, the STING agonist introduced into the cell by the ISAC molecule at this concentration may not be sufficient to activate the STING pathway, resulting in a reduced killing effect of ISAC compared with that of a naked mAb.

In vivo experiments revealed the crucial role of the host STING pathway in B002T‐LP004 efficacy. Tumor growth was significantly inhibited in mice with normal host STING when treated with B002T‐LP004 (Figure 4C, E). However, in mice with normal tumor STING but knockout of host STING, rapid tumor growth continued despite treatment (Figure 4B). Similarly, in models where both tumor and host STING were knocked out, tumors grew rapidly, showing the highest growth rate among all ISAC‐treated groups (Figure 4D). These findings highlight that the host STING pathway dominates the therapeutic efficacy of STING‐ISAC, while the tumor STING pathway has minimal impact.

Immediately following the administration of B002T‐LP004, flow cytometry revealed rapid initial declines in peripheral blood immune cells (T, NK, NKT, and DC cells), which later recovered to control levels (Figure 5A). This suggests immune cells were recruited from blood to tumors, driving antitumor effects. Conversely, tumor tissue showed increased CD45⁺ immune cells but decreased T, B, NK, and NKT cells (Figure 5B). Analysis also showed increased activation of immune cells in both blood and tumors, indicating B002T‐LP004's ability to mobilize and activate these cells (Figure S4A, C).

Furthermore, the impact of B002T‐LP004 on cytokines expression was investigated. Figure S4B, D illustrates the alterations in cytokine profiles in hormonal model mice following the administration of B002T‐LP004. Cytokines, including IFN‐γ, KC, TNF‐α, MCP‐1, IL‐12p70, IP‐10, and IFN‐α, in the peripheral blood was significantly greater than that in the control group. This increase was most pronounced between 3 and 12 h, with a subsequent decrease to a level approaching that of the control group at 168 h. The alterations in cytokine levels within the tumor tissue exhibited a comparable trend to those observed in the peripheral blood.

To identify the immune cells involved in B002T‐LP004‐mediated STING activation and antitumor effects, knockout experiments were performed in C57BL/6J mice. Results (Figure 6A, B) showed that removing any single immune cell type did not affect B002T‐LP004's ability to induce complete tumor regression. Further experiments in severely immunodeficient NCG mice (Figure 6C) demonstrated that B002T‐LP004 remained effective even with only DCs and macrophages, though complete tumor regression was not achieved. These findings indicate that ISACs mobilize multiple immune cell types, rather than relying on a single type, to destroy tumors.

Finally, the safety of B002T‐LP004 was also evaluated. The liver and kidney functions of the tumor‐bearing mice were examined, and the results are presented in Figures 7 and S5. These figures demonstrate that the liver and kidney functions of the mice that received B002T‐LP004 were restored to levels comparable to those of healthy mice. This result suggests that B002T‐LP004 has an acceptable safety profile.

The development of agonist conjugates is difficult, since it is hard to delineate the degree of activation of the immune system, and to assess exactly how large the differences are among different patients. This results in a narrow window for the safe use of ISACs, which might be one of the factors that have hindered clinical trials. Despite of the current winding research and development, agonist‐based conjugated drugs will be a highly promising development direction in the future for low‐response or drug‐resistant tumor diseases.

## 4 Conclusion

In summary, our research has developed the next‐generation ISAC targeting HER‐2 tumors. Compared with existing similar ISACs, it has higher DAR values and better stability. In the four constructed tumor models in mice, it shows excellent in vivo efficacy targeting tumors with high and low HER2 expression, and HER2 resistance. The long‐term immune memory of ISAC enables it to respond rapidly when re‐invaded by the same tumor. This study provides new research directions and options for the long‐standing HER2 tumor drug resistance and promoting the development of new ISACs drugs and clinical application of STING agonists.

## 5 Materials and Methods

### 5.1 Linker‐STING Agonist

LP‐004 (the synthetic information is detailed in the patent, application number: CN 118619928 A) was developed by our team.

### 5.2 Expression and Purification of the Anti‐HER2 Antibody B002T

According to the amino acid sequence of trastuzumab (a recombinant anti‐HER2 monoclonal antibody, https://go.drugbank.com/drugs/DB00072), the DNA sequences of the light chain and heavy chain were synthesized and cloned and inserted into the pCHO1.0 expression vector. The recombinant pCHO1.0‐HER2 plasmid was transfected into CHO‐S host cells via the lipofection method to establish a stable cell line with the selection drugs puromycin (A1113803; Gibco) and MTX (M8407; Sigma). Positive clones were screened to obtain a final monoclonal cell line by limit dilution on the basis of cell growth, expression and product quality. To produce antibodies, the seeded cells were cultured in Hycell CHO medium (SH30934.01; Cytiva) for resuspension and expanded to a density greater than 3.5×106 cells/mL. They were transfected and cultured in a 30 L bioreactor containing Dynamis medium (A24363SA; Gibco) for 14 days. EfficientFeed C+ media supplemented with AGT was added starting from the 3rd day. The collected cell culture supernatant was purified into an antibody‐drug stock solution via protein A affinity chromatography, low pH inactivation, anion chromatography, cation chromatography, viral filtration, and ultrafiltration/diafiltration steps.

### 5.3 Preparation of the STING Agonist ISAC: B002T‐LP004

A solution of 20 mg of anti‐HER2 monoclonal antibody (*c* = 10 mg/mL, dissolved in pH = 7.4 PBS) was prepared, followed by the addition of triple molar equivalents of TCEP. The reduction was conducted at 0°C for 24 h. The LP‐004 molecule (10 equivalents) was dissolved in DMA and added to the above solution. The coupling was continued at room temperature for 2 h. At the end of the reaction, the monoclonal antibody was subjected to centrifugation. The solution was then purified by elution using an Amicon Ultra 15 mL (50 kDa) and an Akta Avant 150. The elution system consisted of citrate buffer as follows: citrate dihydrate (5.59 mg), citrate monohydrate (0.21 g), sucrose (60 g), and purified water to a total mass of 1 kg.

### 5.4 Determination of the Drug‐to‐Antibody Ratio (DAR) in the STING Agonist ISAC

Samples of ISAC were taken in 1.5 mL EP tubes and diluted to 100 µL with PBS, resulting in a final concentration of 4 mg/mL. To the diluted sample, 100 µL of 8 M guanidine hydrochloride solution and 50 µL of 0.5 mL of DTT solution were added, and the mixture was incubated in a water bath at 37°C for 30 min. Centrifugation at 12,000 rpm for 5 min was then performed, after which 200 µL of the supernatant was removed from the injection vials for the uptake assay. A Waters ACQUITY UPLC was utilized with mobile phases A (0.1% FA, H_2_O) and B (0.1% FA, ACN). The gradient elution program was as follows: 0–5 min, 22% B; 5–5.1 min, 22–26% B; 5.1–16 min, 26–36% B; 16–17.5 min, 36–90% B; 17.5–18 min, 90–22% B; and 18–20 min, 22% B. The flow rate was set at 0.35 mL/min, a MAbPac column (4 µm, 2.1 mm × 50 mm (P/N 088648)) was used, the column temperature was 80°C, and 5 µg sample was loaded. A Waters Time‐of‐flight (TOF) mass spectrometer (Synapt G2) equipped was used for mass measurement. The MS settings: resolution mode; capillary voltage, 3.0 kV; cone voltage, 60 V; desolvation temperature, 300°C; ion source temperature, 100°C; the flow rate of drying gas was 800 L/h; the flow rate of cone gas was 50 L/h.

### 5.5 Cell Lines

SK‐BR‐3 human breast cancer cells were purchased from the National Collection of Authenticated Cell Culture and were cultured in McCoy's 5A (Modified) medium supplemented with 10% fetal bovine serum (FBS). Both MC38‐hHER2 (mouse colorectal cancer cells with knockdown of the mouse Her2 gene and overexpression of the human Her2 gene in MC38 cells) and MC38‐hHER2‐STNG KO (mouse colorectal cancer cells with knockdown of the mouse HER2 gene and STING gene and overexpression of the human HER2 gene in MC38 cells) were constructed by Shanghai Jiaolian Drug Development Co. Ltd. Both MC38‐hHER2 and MC38‐hHER2‐STNG KO cells were cultured in MEM/high medium containing 10% FBS supplemented with 1.5 µg/mL blasticidin S HCl (#ST018; Beyotime) and 1 µg/mL puromycin. All the cell lines were maintained at 37°C in 5% CO_2_.

### 5.6 Binding Activity of STING Agonist ISAC to FcγR/HER2 Antigen on the Cell Surface

CHO‐K1/CD16A‐158F cells (GenScript) expressing the FcγRIIIA‐158F receptor, CHO‐K1/CD16A‐158 V cells (GenScript) expressing the FcγRIIIA‐158 V receptor and KYSE‐410 cells (ATCC) expressing the HER‐2 antigen were selected for analysis. Following centrifugation, the cells were resuspended and washed once with DPBS. After resuspension with DPBS again, the cell density was adjusted to 2E^6^ cells/mL, and 100 µL of cell suspension was added to each well of a 96‐well plate. The ISAC samples of varying concentrations were added separately and incubated at 4°C for 1 h. The plate was then washed three times with DPBS, and all the supernatants were removed. Subsequently, the Alexa FluorTM 488‐conjugated goat anti‐human IgG (H+L) secondary antibody (Invitrogen) was diluted 1:400 and added to each well (100 µL per well). The plate was then incubated at 4°C for 40 min. Following two washes with DPBS, the cells were resuspended in 400 µL of DPBS per well, and the mean fluorescence intensity (MFI) was analyzed via flow cytometry.

### 5.7 Real‐time Binding and Analysis by Surface Plasmon Resonance (SPR)

Surface plasmon resonance analysis. Real‐time binding and analysis by surface plasmon resonance (SPR) were conducted on a BIAcore T200 instrument (GE Healthcare) at 25°C. Anti‐human lg (Fc) was diluted with acetate solution (pH = 5.0) to 20 µg/mL and immobilized on channels 1 and 2 of a research‐grade CM5 chip via the amino coupling method (channel 1 of which was the reference channel). The ligands (B002T, B002T‐LP004) were diluted to 2 µg/mL and injected into two channels (sample channels) at a flow rate of 20 µL/min. The ligands were captured by anti‐human IgG (Fc) with a capture time of 60 s, and then the human HER2 antigen as an analyte was injected into channels 1 and 2 at concentrations of 6.25 nM, 12.5 nM, 25 nM, 50 nM and 100 nM at a flow rate of 20 µL/min. The binding time was 180 s, and the dissociation time was 480 s. The chip was regenerated with 3 M MgCl_2_; the regeneration time was 30 s, and the flow rate was 30 µL/min. Finally, the affinities of the B002T, B002T‐LP004 and human HER2 antigens were obtained via a 1:1 binding assay.

### 5.8 Endocytosis of STING Agonist ISAC Drugs

Endocytosis by KYSE‐410 (ATCC), SK‐BR‐3 (ATCC), and THP‐1 (ATCC) cells was detected using flow cytometry. The cells were made into a single cell suspension with a density of 4×10^6^ cells/mL. 200 µL of cell suspension was added to 200 µL of ISAC samples of different concentrations and incubated at 4°C for 60 min to bind antigen on cell surface. The cells were washed 2–3 times with 4°C DPBS to remove unbound antibodies. Cells were resuspended by 650 µL incubation buffer (serum‐free basal medium), and then divided into six groups (100 µL per group), including incubation at 37°C for 2, 4, and 6 h and at 4°C for 2, 4, and 6 h. At the end of the incubation period, 300 µL of 4°C buffer was added to each sample to terminate internalization. The cells were then precipitated by centrifugation at 200 × *g* for 5 min at 4°C, and the supernatant was discarded. Subsequently, the Alexa FluorTM 488‐conjugated goat anti‐human IgG (H+L) secondary antibody (Invitrogen) was diluted 1:400 and added to each well (100 µL per well). The plate was then incubated at 4°C for 30 min. Following two washes with DPBS, the cells were resuspended in 400 µL of DPBS per well and MFI was assayed via flow cytometry. The internalization rate of the samples was calculated by comparing the MFI at 4°C and 37°C.

### 5.9 ADCC Evaluation With Human Peripheral Blood Mononuclear Cells (PBMCs)

Antibody‐dependent cell‐mediated cytotoxicity (ADCC) was evaluated using PBMCs derived from a donor as effector cells and SK‐BR‐3 cells as target cells. PBMC were resuscitated and added to 100 U/mL IL2 (#S10970058; Jiangsu Kingsley Pharmaceutical Co., Ltd.) containing T‐cell medium (#BC‐M‐041; Bio‐Channel) and activated with 5 µg/mL anti‐CD3 antibody (#16‐0037‐85; Invitrogen) and 5 µg/mL anti‐CD28 (#16‐0289‐85; Invitrogen) antibody for 72 h before cell spreading. The effector cells and the target cells (1 × 10^4^ cells) were incubated with each substance, and the effector:target (E:T) ratios used were 10:1, 5:1, 2:1, and 1:1. The samples were subsequently incubated with different drugs. After 48–72 h of incubation, cell viability was evaluated via a CellTiter‐Glo Luminescent Cell Viability Assay (#G7558; Promega) following the manufacturer's instructions.

### 5.10 ADCC Evaluation ISAC With Reporter Bioassay

The density of target cells (KYSE‐410/SK‐BR‐3) was adjusted to 2.5E^5^/mL, and 100 µL of the cell suspension was added to a 96‐well plate and incubated overnight at 37°C in a 5% CO₂ incubator. The samples to be tested were diluted to varying concentrations, and 25 µL was added to a white 96‐well plate and coincubated with cells for 45 min. The effector cells (Rhinogen ADCC Reporter Bioassay) were adjusted to a concentration of 3E^6^/mL, and 25 µL of the cell suspension was added to the 96‐well plate, which was then incubated at 37°C in a 5% CO₂ incubator. After removing the medium from the plate, wash once with ADCC buffer and add 25 µL/well buffer. The 96‐well plate was then incubated for an additional 6 h. The plate was subsequently removed and allowed to equilibrate at room temperature for 15 min. 75 µL of the luciferase assay reagent in the Bioassay was then added, and the signal was immediately detected.

### 5.11 Reporter Gene Assay for STING Activation in THP‐1‐ISG‐luc Cells

The density of the SK‐BR‐3 cells was adjusted to 1E^5^/mL, and 100 µL of the SK‐BR‐3 cell suspension was added to a 96‐well plate and incubated overnight at 37°C in a 5% CO₂ incubator. After removing the medium from the plate, the samples to be tested were diluted to varying concentrations, and 50 µL was added to a 96‐well plate and coincubated with SK‐BR‐3 cells for 30 min. The THP‐1‐Lucia‐ISG cells (Invivogen, thpl‐isg) were adjusted to a concentration of 2E^6^/mL, and 50 µL of the THP‐1 cell suspension was added to a 96‐well plate, which was then incubated at 37°C in a 5% CO₂ incubator. The 96‐well plate was then incubated for an additional 24 h. The plate was subsequently removed and allowed to equilibrate at room temperature for 15 min. Guassia luciferase assay reagent (Invivogen, rep‐qlc4lg1) was then added, and the signal was immediately detected.

### 5.12 Flow Cytometry (FCM)

Blood sample processing: red blood cell lysate (#00‐4300‐54; Bioscience) was added to the blood sample and processed into a single cell suspension, followed by FCM analysis. Lymph node and spleen samples were also processed into single cell suspensions, then FCM analysis was performed.

The tumor samples were digested with enzymes (#DHTE‐5001; RWD) and processed with the tissue processor (#DSC‐800; RWD). The treated solution was filtered through a cell strainer (70 µm) to obtain single cell suspensions, then FCM analysis was performed.

For surface staining, the single cell suspensions were incubated with Fc blocking (#156604; Biolegend) for 10 min at 4°C and subsequently stained with an antibody cocktail against CD11b (#550993; BD), CD11C (#117339; Biolegend), CD45 (#103140; Biolegend), CD80 (#104708; Biolegend), CD86 (#105032; Biolegend), CD206 (#25‐2061‐82; eBioscience), CD274 (#124324; Biolegend), F4/80 (#17‐4801‐82; eBioscience), and MHCII (#107645; Biolegend) in the dark at 4°C for 1 h and then washed with FACS buffer. Sytox Blue (#S34857; Invitrogen) was then added, and the mixture was incubated for 5‒10 min before detection.

For intracellular staining, the single‐cell suspensions were incubated with Fixable Viability Dye eFluor 780 (#65‐0865‐18; eBioscience) in the dark at 4°C for 30 min and then washed with FACS buffer. The single‐cell suspensions were incubated with Fc block (#156604; Biolegend) for 10 min at 4°C and subsequently stained with an antibody cocktail against CD3 (#100216; Biolegend), CD3 (#555274; BD), CD4 (#100414; Biolegend), CD4 (#100447; Biolegend), CD8 (#100734; Biolegend), CD8 (#562283; BD), CD11b (#550993; BD), CD11C (#117339; Biolegend), CD19 (#115546; Biolegend), CD45 (#103140; Biolegend), CD69 (#104507; Biolegend), CD80 (#104743; Biolegend), CD86 (#105110; Biolegend), CD107a (#121618; Biolegend), CD274 (#124324; Biolegend), NK1.1 (#550627; BD), and MHCII (#107645; Biolegend) in the dark at 4°C for 1 h. The samples were fixed in fixation buffer (#00‐8222‐49; eBioscience) at room temperature for 50 min, washed with permeabilization/wash buffer (#00‐8333‐56; eBioscience), and incubated.

### 5.13 Pharmacodynamic (PD) Analysis

Whole blood was collected from the mice, and the serum was isolated for cytokine detection. Tumor tissue samples were lysed with lysis buffer (#78510; Thermo Scientific), and the lysed supernatants were used for cytokine detection. Indicators of the cytokine detection profile included IFN‐γ, CXCL1 (KC), TNF‐α, CCL2 (MCP‐1), IL‐12p70, CCL5 (RANTES), IL‐1β, CXCL10 (IP‐10), GM‐CSF, IL‐10, IFN‐β, IFN‐α, and IL‐6 (#740622; Biolegend), and the detection was conducted according to the LEGENDplex kit instructions.

### 5.14 In Vivo Efficacy Experiments

HCC1954, SNU5, JIMT‐1, and Mc38‐hHER‐2 tumor cells were selected to construct different transplantation tumor models in CB17/SCID and C57/BL6 mice. Eight‐week‐old mice were selected and inoculated with 1 × 10^7^ tumor cells. Once the tumor has reached an appropriate size (average volume 80–120 mm^3^), ISAC administration is performed, and subsequent changes in tumor volume, body weight and other indicators are observed. In the tumor rechallenge experiments, the drug was administered on a single occasion, and the complete disappearance of the tumor was ensured before each subsequent administration.

### 5.15 Generation of STlNG‐Knockout MC38‐hHER2‐STNG‐KO Cells

CRISPR/Cas9‐mediated gene knockout was used to generate MC38‐hHER2‐STNG‐KO cells. MC38‐hHER2 cells were transfected with the Lenti‐CRISPR‐Cas9‐sting·sgRNA plasmid (sg#1: acggcccagaggtcaccgct, sg#2: ggtcatactacattgggtac) via ExFect Transfection Reagent (Vazyme, cat. T101‐AA). Selection was started by adding puromycin (Thermo Fisher) to the cell culture media approximately 24 h after transfection. Single cells were then plated in individual wells of a 96‐well plate, and clones were identified via PCR sequencing. STING knockout cells were designated MC38‐hHER2‐STNG KO. The MC38‐hHER2‐STNG‐KO cell lines selected according to the sequencing results were expanded and cultured, and cells with good growth status were selected and plated on white bottom transparent 96‐well plates overnight. The cells were transfected with the pGL4.21/ISG5 plasmid via ExFect Transfection Reagent. After 24 h of transfection, MCB‐22‐049 (sting agonist) at a final concentration of 100 nM was added for stimulation, and the cells were incubated at 37°C and 5% CO_2_ for 6 h. A biolite luciferase assay system (Vazyme, cat. DD1201‐02) was added, and the chemiluminescence value was detected.

### 5.16 In Vivo Efficacy Experiments in STING‐KO Mice

In vivo efficacy studies were conducted in severe combined C57BL/6 and C57BL/6J‐STING‐KO mice (Gempharmatech). We suspended 3 × 10^6^ MC38‐hHER2 or MC38‐hHER2‐STNG KO cells in 0.1 mL of PBS (pH 7.4) and injected them subcutaneously into the right flanks of the mice. For the colon cancer MC38‐hHER2 and MC38‐hHER2‐STNG KO xenograft experiments, treatment was initiated when the tumor volume averaged 80–120 mm^3^ and was administered once intravenously at an ISAC concentration of 2 or 0.075 mg/kg (i.v., 10 µL/g, *n*  = 4–8 mice per group). Tumor volumes were measured twice a week via a caliper device (length × width) and calculated via the following formula: TV = 0.5 × *a* × *b* ^2^, where *a* and *b* are the long and short diameters of the tumor, respectively. When the tumor volume of an individual mouse is greater than 3000 mm^3^, the mouse will be euthanized, and photos of the mouse and tumor will be taken.

### 5.17 In Vivo Immune Cell Depletion

NK cells were depleted during MC38‐hHER2 tumor growth via the intraperitoneal injection of 100 µL of anti‐asialo GM1 (#146002; BioLegend) or 1 mL of water to dissolve asialo GM1 powder, 20 µL of which was taken and diluted in PBS to 100 µL via intraperitoneal injection into the mice (Q5D × 5 doses). CD4+ T cells and CD8+ T cells were depleted via InVivoMAb anti‐mouse CD4 (#BE003‐1; BioxCell) and InVivoMAb anti‐mouse CD8 (#BE0061; BioxCell) antibodies and intraperitoneal injections of 200 µg/mouse, Q4D × 6 doses. The macrophages were depleted of clodronate liposomes (#40337ES10 and #40337ES08; Yeasen) at a dose of 1 mg/mouse, Q4D × 6.

### 5.18 Safety Evaluation

Four C57BL/6J mice were subcutaneously inoculated with MC38‐hHER2 cells. When the average tumor volume reached 113.17 mm^3^, the mice were grouped. The day of grouping was designated as D0, and dosing was initiated. The administration volume was 5 µL/g × body weight (g). Subsequently, tumor size was measured twice weekly, and body weight was recorded twice weekly. At the experimental endpoint (D24), mice were photographed, tumors were excised, weighed, and photographed, approximately 100 µL of peripheral blood was collected for flow cytometry, and approximately 60 µL of serum was collected for ALT, AST, CREA, and BUN measurements.

### 5.19 Statistical Analysis

In vitro experiments were performed in triplicate for each group, and the data are presented as the mean ± standard error of the mean (SEM). IC_50_ and EC_50_ values were calculated via GraphPad Prism 9 software, which was also used for data visualization. Survival data are expressed as the mean ± standard deviation (SD), and additional data are expressed as the mean ± SEM. For comparisons between two groups of samples, independent t tests were performed. All the data were analyzed via SPSS (version 24). *p* < 0.05 was considered statistically significant.

## Author Contributions

Research design: Junzhi Wang and Ao Zhang. Data collection: Gang Wu, Shengtao Yao, Zhihao Fu, and Yu Liu. Data analysis: Gang Wu and Shengtao Yao. Manuscript preparation: Shengtao Yao and Guanghao Wu. Manuscript editing: Shengtao Yao, Jialiang Du, Yiming Fan, Chuanfei Yu, and Chunyong Ding. All the authors have read and approved the final manuscript.

## Ethics Statement

All studies in this paper comply with all relevant ethical regulations. This institution is accredited by the International Laboratory Animal Evaluation and Accreditation Management Committee (AAALAC International) and has an animal use license (SYXK (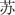) 2023‐0036, approved by the Animal Management Committee of the Jiangsu Provincial Department of Science and Technology).

The protocol and any amendments or procedures involving the care and use of animals in this study were reviewed and approved by the Institutional Animal Care and Use Committee (IACUC) of GemPharmatech Co., Ltd. before the study was conducted, and the Animal Protocols (AP) numbers are GPTAP20231026‐5, GPTAP20231222‐2, GPTAP20240202‐6, and GPTAP20240529‐3643 respectively. During the study, the care and use of the animals were conducted in accordance with the regulations of the Association for Assessment and Accreditation of Laboratory Animal Care (AAALAC).

## Conflicts of Interest

Authors Shengtao Yao, Yu Liu, and Guanghao Wu are employees in Shanghai SPH Jiaolian Pharmaceutical Technology Co., Ltd., Shanghai, China, but have no potential relevant financial or nonfinancial interests to disclose. The other authors have no conflicts of interest to declare.

## Supporting information



Supporting Information

## Data Availability

All datasets generated and analyzed during this study are included in this published article and its Supplementary Information files. Additional data are available from the corresponding author on reasonable request.
