# Supplementary material for: Next‐Generation HER‐2 Tumor‐Targeted Delivery of the STING Agonist Immune‐Stimulating Antibody Conjugate (ISAC) Improves Anticancer Efficacy and Induces Immunological Memory
Source: MedComm (2020). 2025 Jul 2;6(7):e70254. doi: 10.1002/mco2.70254 (PMC12214944; doi:10.1002/mco2.70254)
Supplement: Supplementary file 1 — Supporting Information [file MCO2-6-e70254-s001.docx]

**Next-generation** **HER-2 tumor-targeted delivery of the STING agonist immune-stimulating antibody conjugate (ISAC) improves anticancer efficacy and induces immunological memory**

Gang Wu^1,2#^, Chuanfei Yu^2#^, Chunyong Ding^3#^, Shengtao Yao^4^, Jialiang Du^2^, Zhihao Fu^2^, Yu Liu^4^, Yiming Fan^3^, Guanghao Wu^4^, Ao Zhang^3*^, Junzhi Wang^1^^*^

^1^School of Life Science and Biopharmaceutics, Shenyang Pharmaceutical University, No. 103 Wenhua Road, Shenyang 110016, China.

^2^ National Institutes for Food and Drug Control, State Key Laboratory of Drug Regulatory Science, NHC Key Laboratory of Research on Quality and Standardization of Biotech Products, NMPA Key Laboratory for Quality Research and Evaluation of Biological Products, Beijing, 102629, China.

^3^Shanghai Frontiers Science Center of Drug Target Identification and Delivery, and National Key Laboratory of Innovative Immunotherapy,School of Pharmaceutical Sciences, Shanghai Jiao Tong University, Shanghai 200240, China.

^4^ Shanghai SPH Jiaolian Pharmaceutical Technology Co., Ltd., Shanghai, 200000, China.

*Corresponding authors. Email addresses: wangjz@nifdc.org.cn; [ao6919zhang@sjtu.edu.cn](mailto:ao6919zhang@sjtu.edu.cn)

^#^ These authors contributed equally to the work.

**Manuscript Supporting Information (SI)**

**Table of Contents**

Supporting information 1: ……………………………………..S2

Supporting information 2: ……………………………………..S8

Supporting information 3: ……………………………………..S9

Supporting information 4: ….……………………………..…...S10

Supporting information 5: ……………………..………………S14

Supporting information 6: ……………………..………………S15

**Supporting information 1:**

**Theoretical conjugation forms, DAR determination and stability in plasma analysis of ISACs**

*
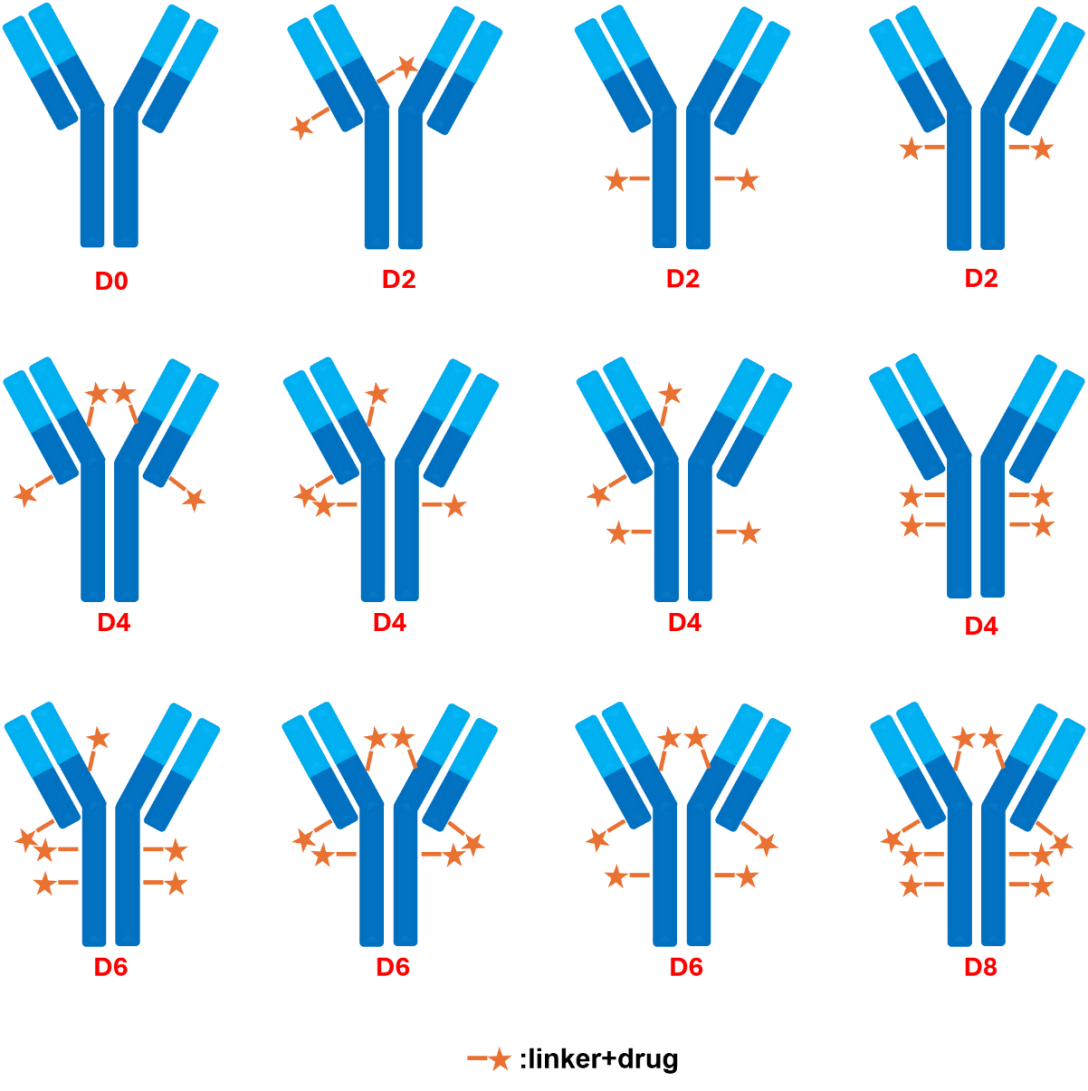
*

**Figure S1A. Schematic diagram illustrating all the theoretical conjugation forms of B002T-LP004 (D0 to D8).**


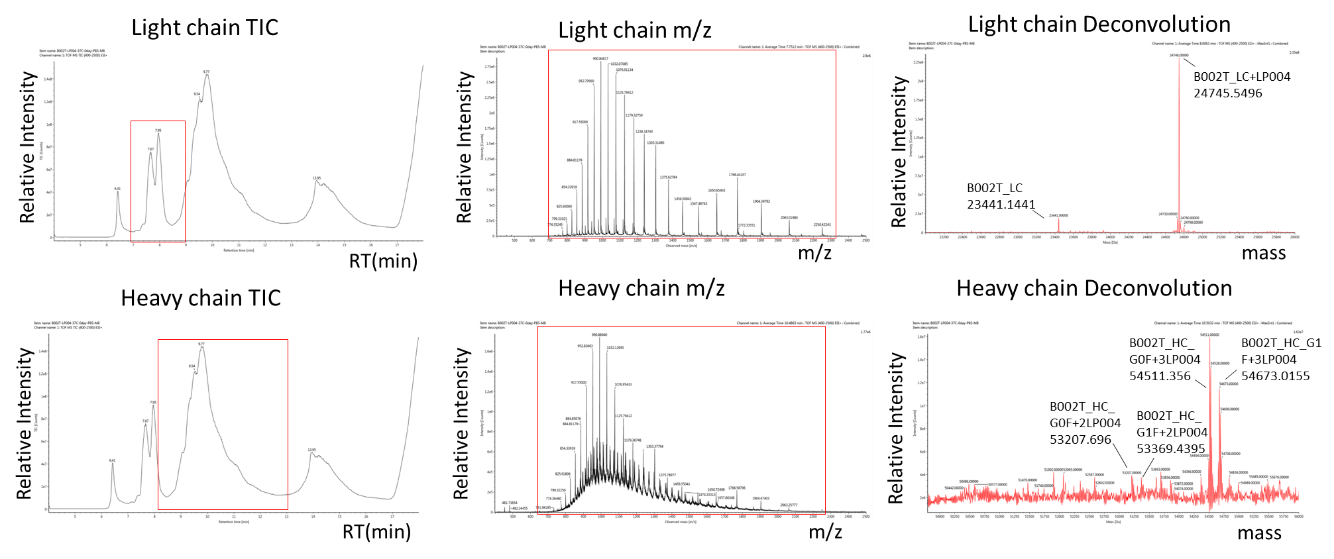


**Figure S1B. BPC profiles, full MS spectra and deconvolution results of the reduced chains of B002T-LP004.**

**Figure S1C. The stability of B002T-LP004 was investigated in PBS and plasma.**


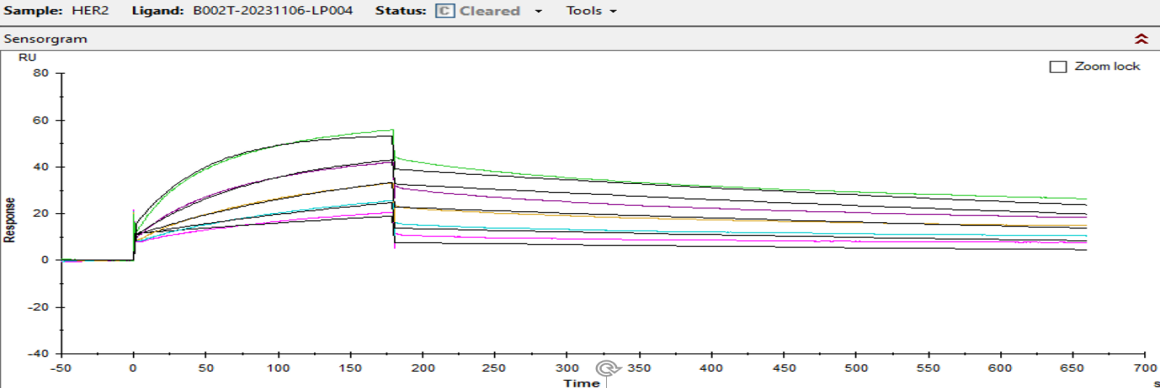


**Figure S1D. The affinity between the Fabs of B002T-LP004 and the antigen was determined via surface plasmon resonance (SPR).**


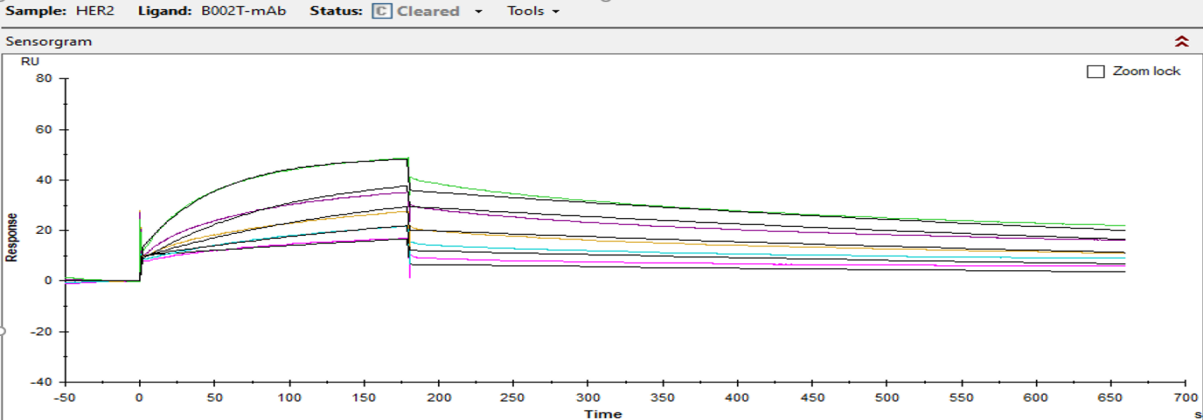


**Figure S1E. The affinity between Fabs of B002T (naked mAb) and the antigen was determined via the surface plasmon resonance (SPR) method.**

**Figure S1F. Internalization of the B002T-LP004 in SKBR3 cell.**

**Figure S1G. Internalization of the B002T-LP004 in THP1 cell.**

**Figure S1H. ADCC effect assay of the B002T-LP004 in SK-BR3 cell.**

**Figure S1I. Flow cytometry assay for the activity of B002T-LP004 in the coculture assay with PBMCs in vitro.**

**Figure S1J. The activity of B002T-LP004 at 10 mg/mL in *in vitro* coculture assays with SK-BR/PBMC ratios ranging from 10:1-1:1.**

**Figure S1K. Lucia-ISG assay of B002T-LP004 in THP-1 cells.**

**Supporting information 2:**

**Antitumor effects** **and rechallenge study of ISAC in an MC38 hHER2/C57 model**

**Figure S2A. Antitumor effects of B002T-LP004 in the MC38 hHER2/C57 mouse model.**

活化Tc

活化Th

活化NK&NK T

活化DC

活化Tc

活化Tc

活化NK&NK T

活化NK&NK T

活化DC

活化DC

活化NK T

活化NK T

活化NK T

**Figure S13**

**Figure S2B. Rechallenge antitumor effects of B002T-LP004 in a B16F10/C57 mouse model**

**Supporting information 3:**

Generation of STlNG-knockout MC38-hHER2-STNG-KO cells


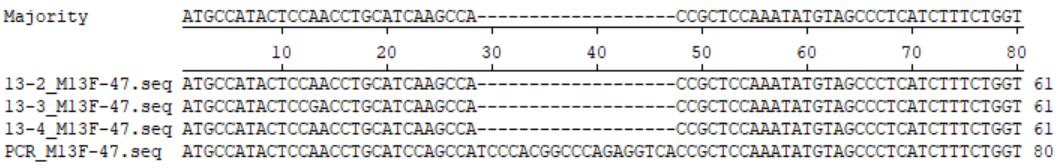


**Figure S3A.MC38-hHER2-STING-KO cells were iendtified via PCR**

**Figure S3B. MC38-hHER2-STNG-KO cells were identified via a luciferase-based cell-based sting activation assay.**

**Supporting information 4:**

Cytokine and immune cell activation in plasma and tumor cells was analyzed after ISAC administration.

**Figure S4A. Flow cytometric assay to determine the activation of different types of immune cells in peripheral blood by ISACs.**

**G1: Vehicle; G2: B002T, 2 mpk; G3: LP004, 0.5 mpk; G4: B002T-LP004, 2 mpk.**

**Figure S4B. Changes of cytokine content in serum. G1: Vehicle; G2: B002T, 2 mpk; G3: LP004, 0.5 mpk; G4: B002T-LP004, 2 mpk.**

**Figure S4C. Flow cytometric assay to determine the activation of different types of immune cells in tumor tissue by ISACs. G1: Vehicle; G2: B002T, 2 mpk; G3: LP004, 0.5 mpk; G4: B002T-LP004B002T-LP004, 2 mpk.**

**Figure S4D. Changes of cytokine content in serum. G1: Vehicle; G2: B002T, 2 mpk; G3: LP004, 0.5 mpk; G4: B002T-LP004, 2 mpk.**

**Supporting information 5:**

The hepatic and renal function datas from healthy mice.

**Figure S5.** **Indicators of hepatic and renal function in healthy mice.**

**(A): AST; (B) ALT; (C) BUN; (D) CREA.**

**Supporting information 6:**

**Table S1. Grouping of mouse models and design of dosing regimens in mechanistic studies**

| **Group** | **No. of mice** | **Mice strain** | **Cell lineage** | **Drug name** | **Dose**  **(mg/kg)** | **Route of Administration** | **Frequency of administration** |
| --- | --- | --- | --- | --- | --- | --- | --- |
| **G1** | **8** | **C57BL/6J -STING-KO** | **MC38-hHer2** | **Vehicle** | **-** | ***i.v.*** | **Once** |
| **G2** | **8** | **C57BL/6J -STING-KO** | **MC38-hHer2** | **ISAC**  **(B002T-LP004)** | **2** | ***i.v.*** | **Once** |
| **G3** | **8** | **C57BL/6J** | **MC38-hHER2-STING-KO** | **Vehicle** | **-** | ***i.v.*** | **Once** |
| **G4** | **8** | **C57BL/6J** | **MC38-hHER2-STING-KO** | **ISAC**  **(B002T-LP004)** | **2** | ***i.v.*** | **Once** |
| **G5** | **4** | **C57BL/6J -STING-KO** | **MC38-hHER2-STING-KO** | **Vehicle** | **-** | ***i.v.*** | **Once** |
| **G6** | **4** | **C57BL/6J -STING-KO** | **MC38-hHER2-STING-KO** | **ISAC**  **(B002T-LP004)** | **2** | ***i.v.*** | **Once** |
| **G7** | **4** | **C57BL/6J** | **MC38-hHer2** | **Vehicle** | **-** | ***i.v.*** | **Once** |
| **G8** | **4** | **C57BL/6J** | **MC38-hHer2** | **ISAC**  **(B002T-LP004)** | **2** | ***i.v.*** | **Once** |

**Table S2. The specific markers for the flow cytometry assay.**

| **Panel 1** | | **Panel 2** | | **Panel 3** | |
| --- | --- | --- | --- | --- | --- |
| **Marker** | **Color** | **Marker** | **Color** | **Marker** | **Color** |
| **mCD45** | **BV605** | **mCD45** | **BV605** | **mCD45** | **BV605** |
| **mCD3** | **AF700** | **mCD3** | **BV711** | **mCD11b** | **p55** |
| **mCD4** | **APC-H7** | **mCD4** | **FITC** | **mCD11c** | **BV650** |
| **mCD8** | **P55** | **mCD8** | **PE-D594** | **mCD80** | **PE** |
| **mCD69** | **PE** | **mCD69** | **PE** | **mPDL1** | **PE-D594** |
| **mGzmB** | **PE-cy7** | **GranzymeB** | **PE-cy7** | **F4/80** | **APC** |
| **mCD107a** | **BV421** | **mCD107a** | **BV421** | **mCD206** | **PE-cy7** |
| **mNK1.1** | **APC** | **mNK1.1** | **APC** | **mCD86** | **BV421** |
| **mCD11c** | **BV650** | **mCD19** | **BV510** | **MHCⅡ** | **BV785** |
| **mIA/IE** | **BV785** | **CD11b** | **P55** | **D/L** | **BV510** |
| **mCD80** | **BV711** | **D/L** | **ef780** |  |  |
| **mCD86** | **FITC** |  |  |  |  |
| **mCD274** | **PE-D594** |  |  |  |  |
| **D/L** | **ef506** |  |  |  |  |

**Table S3. Mouse models were grouped, and dosing regimens were designed after immune cells (CD4+ T cells, CD8+ T cells, NK cells, and macrophages) were removed.**

| **Group** | **Number of mice** | **Treated** | **Dose** | **Delivery route** | **Frequency** |
| --- | --- | --- | --- | --- | --- |
| **G1** | **4** | **Vehicle** | **-** | ***i.v.*** | **Once** |
| **G2** | **4** | **ISAC(B002T-LP004)** | **2mpk** | ***i.v.*** | **Once** |
| **G3** | **4** | **Vehicle** | **-** | ***i.v.*** | **Once** |
|  |  | **anti-mCD4 antibody** | **200 μg** | ***i.p.*** | **Q4D × 6 doses** |
| **G4** | **4** | **ISAC(B002T-LP004)** | **2 mpk** | ***i.v.*** | **Once** |
|  |  | **anti-mCD4 antibody** | **200 μg** | ***i.p.*** | **Q4D × 6 doses** |
| **G5** | **4** | **Vehicle** | **-** | ***i.v.*** | **Once** |
|  |  | **anti-mCD8a antibody** | **200 μg** | ***i.p.*** | **Q4D × 6 doses** |
| **G6** | **4** | **B002T-LP004** | **2 mpk** | ***i.v.*** | **Once** |
|  |  | **anti-mCD8a antibody** | **200 μg** | ***i.p.*** | **Q4D × 6 doses** |
| **G7** | **4** | **Vehicle** | **-** | ***i.v.*** | **Once** |
|  |  | **anti-asialo GM antibody** | **20μl** | ***i.p.*** | **Q5D × 5 doses** |
| **G8** | **4** | **ISAC(B002T-LP004)** | **2 mpk** | ***i.v.*** | **Once** |
|  |  | **anti-asialo GM antibody** | **20μl** | ***i.p.*** | **Q5D × 5 doses** |
| **G9** | **4** | **Vehicle** | **-** | ***i.v.*** | **Once** |
|  |  | **Clodronate Liposomes** | **1 mg** | ***i.p.*** | **Q4D × 6 doses** |
| **G10** | **4** | **ISAC(B002T-LP004)** | **2mpk** | ***i.v.*** | **Once** |
|  |  | **Clodronate Liposomes** | **1 mg** | ***i.p.*** | **Q4D × 6 doses** |
